# Multiple Skin Cancers Associated With Hydroxyurea: Case Report and Review of Literature

**DOI:** 10.1002/cnr2.70304

**Published:** 2025-08-09

**Authors:** Safoura Shakoei, Zahra Nilfroushan

**Affiliations:** ^1^ Department of Dermatology Imam Khomeini Hospital Complex, Tehran University of Medical Sciences (TUMS) Tehran Iran; ^2^ Department of Dermatology Razi Hospital, Tehran University of Medical Sciences (TUMS) Tehran Iran

**Keywords:** Bowen, essential thrombocytopenia, hydroxyurea, review, squamous cell carcinoma

## Abstract

**Background:**

Hydroxyurea (HU) is an antimetabolite used to treat different myeloproliferative disorders, including chronic myeloid leukemia, polycythemia vera, and essential thrombocytopenia (ET). HU has a significant effect on the treatment of myeloproliferative disease.

**Recent Findings:**

A 54‐year‐old woman with a history of ET from 15 years ago presented with multiple erythematous plaques and ulcers on the face and extremities from 2 years ago. She had multiple SCCs, Bowen, and AK on both extremities and face. We reviewed and summarized the literature on the relationship between HU and nonmelanoma skin cancer (NMSC) between 1991 and 2022. Sixty cases (37 men and 23 women) were evaluated. The participants' mean age was 66.73 ± 9.80 years. They received the HU therapy for 9.17 ± 4.29 years. The latency period to the occurrence of symptoms in patients with HU‐related skin cancer was 7.33 ± 4.40 years. The HU dose in these patients ranged from 1.21 ± 0.53 g/d. Among NMSCs, SCCs are more frequently associated with the HU treatment, followed by AKs, BCCs, and Bowen.

**Conclusion:**

Healthcare providers should be aware of skin cancer risks; patients receiving long‐term HU therapy should be screened for early diagnosis and provided with preventive measures and appropriate treatment.

## 1 Introduction

Hydroxyurea (HU) is a potent myelosuppression drug reducing deoxyribonucleotide production [1] and treats chronic myelogenous leukemia (CML), polycythemia vera (PV), and essential thrombocytosis (ET) [2]. HU has some side effects, including dermatologic side effects, especially during long‐term treatment; the most common of which are nail changes, hyperpigmentation, atrophy of the skin, partial alopecia, subcutaneous tissue changes, scaling, hands and face erythema, and less common lower extremity ulceration [3, 4, 5].

The literature reports some cases of HU‐related cutaneous squamous cell carcinoma (SCC) [6, 7, 8]. Accordingly, we reviewed and summarized the literature on the relationship between HU and skin cancer.

This case report presents a unique and compelling narrative of a 54‐year‐old woman who developed multiple skin lesions, including SCC and Bowen's disease, after 14 years of continuous HU therapy. By documenting this patient's clinical progression, treatment challenges, and outcomes, we aim to enrich the existing literature with a comprehensive understanding of the risks posed by prolonged HU therapy.

Unlike prior studies that typically address isolated cases or limited follow‐up periods, our report reviews similar patients and analyzes the effects of HU on skin health over an extended duration.

## 2 Case Report

A 54‐year‐old woman with a history of ET was referred to Razi Hospital (September 2022) with erythematous plaques and ulcers on the face and extremities.

She had undergone splenectomy 14 years ago and was treated with HU at a dose of 1.5 g per day for 14 years. Her medical, family, and psychological history was negative. However, we did not have any information about her genetics. Her first lesions on her right hand were 8 cm × 5 cm, which appeared two years ago. Her lesion was excised with the diagnosis of SCC, and a skin graft was performed. Unfortunately, she had not stopped consuming HU, and her cutaneous changes were ignored.

**FIGURE 1:**
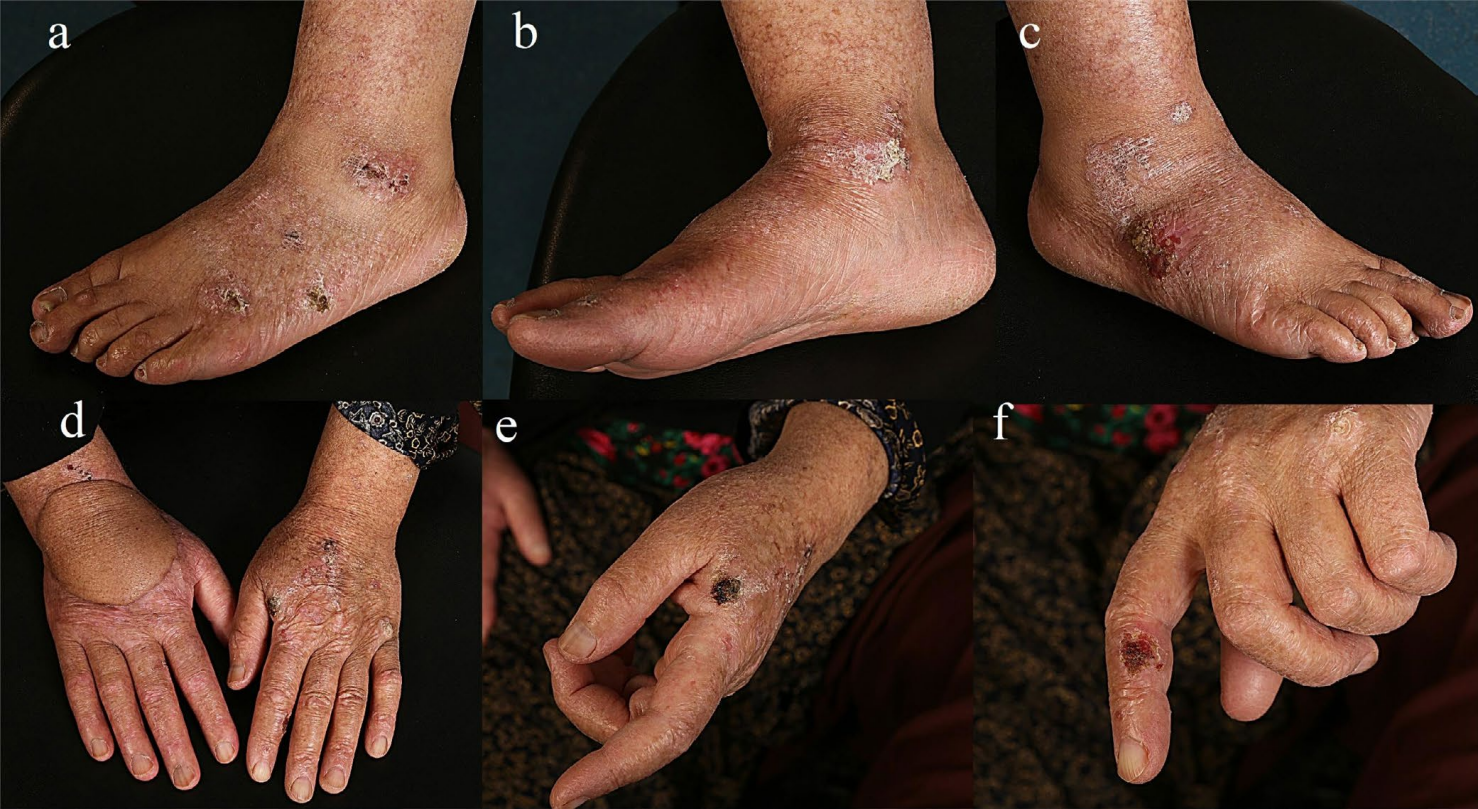
a‐f: Multiple irregulars and ill‐defined scaly red plaques and ulcers developed on her upper and lower extremities.

Physical examinations revealed extensive photodamage and atrophy on her dorsal hands, feet, and face. Multiple irregular and ill‐defined scaly red plaques and ulcers developed on her extremities and face. Hyperkeratotic lesions, branny desquamation, and dotted hyperpigmentation were also noticed on her extremities and face (Figure 1a–f). The size of the lesions ranged from 0.5 to 6 cm.

Several skin biopsies from her right foot and left hand (two lesions) revealed hyperkeratotic and acanthotic epidermis with low‐grade dysplasia, compatible with Bowen. Moreover, the last biopsy from the lower extremity showed a well‐differentiated SCC with 0.6‐mm thickness.

As our incentive to prepare this report, the distinctive feature of this patient was that she had multiple SCCs, Bowen, and AK on both extremities (at least five lesions confirmed pathology before the surgery).

After confirming the diagnosis, the patient was referred to a tumor surgeon; three lesions from the left foot, one lesion from the right foot, one lesion from the left hand, and one from the right hand were resected and grafted. HU was also changed to anagrelide, and she was then treated with an oral retinoid to prevent cancer recurrence. However, six months after stopping HU, a lesion on the patient's right hand recurred, and the tumor involved her underlying bone tissue, which unfortunately led to the amputation of the patient's hand. Complete written informed consent was obtained from the patient for the publication of this study and accompanying images.

## 3 Discussion

This case report highlights several significant findings and challenges associated with long‐term HU therapy, particularly its potential to induce cutaneous malignancies. The most notable findings from our study include the development of multiple skin lesions, specifically SCC and Bowen's disease. This underscores the importance of long‐term dermatological monitoring in patients undergoing prolonged HU therapy, as the cumulative exposure may significantly elevate the risk of skin malignancies.

One of the primary challenges identified in this study is the difficulty in establishing a direct causal relationship between HU use and the development of skin cancers. While previous studies have documented an increased incidence of malignancies in patients treated with HU, there is often a lack of long‐term follow‐up data that comprehensively addresses the specific types of skin cancers that may arise. For instance, a study by Rogers et al. [9] indicated an increased risk of nonmelanoma skin cancers (NMSCs) among patients receiving HU, but it did not provide the timeline of lesion development [9].

NMSC is the most common malignant tumor in fair‐skin people. The primary risk factors for BCCs and SCCs are fair skin and exposure to UVR; other risk factors, especially for SCC, include exposure to ionizing radiation, arsenic or organic chemicals, human papillomavirus infection, immunosuppression, and genetic predisposition. Some drugs are associated with NMSC risks. Long‐term HU therapy can also lead to NMSC [10]. These side effects of HU are rare, and dermatologists should pay attention to this point.

In recent years, the relationship between NMSC and several cases of long‐term HU use has been reported [10, 11, 12]. The HU‐induced skin lesions are often multiple and more common in sun‐exposed areas [3].

HU is converted to free radical nitroxide in vivo and inhibits DNA synthesis. HU inhibits the repair of impaired DNA by radiation [13]. Furthermore, chronic UVB contact induces the clonal expansion of the cp53 mutation in keratinocytes [14].

Due to the high oxidative stress and defects in nucleotide repair caused by HU treatment, chronic damage to basal keratinocytes develops. This damage enables clonal expansion and plays an important role in HU‐associated squamous dysplasia [14].

A literature review evaluated 60 cases (37 men and 23 women), including our patient (Table 1). The participants' mean age was 66.73 ± 9.80 years (range = 45–85 years). The most frequently reported diseases were PV, CML, and ET (46.7%, 26.7%, and 20%).

**TABLE 1:** Summary of the cutaneous non‐melanoma skin cancer of hydroxyurea reported in the literature.

Author	Age	Sex	Disease	Dose of HU (gr/day)	The first lesion appeared (year)	Duration of treatment (year)	Number	NMSC	Site of NMSC
Disdier [15]	65	Man	CML	1	3	N/A	1 BCC, multiple SCC	BCC, SCC	Face, scalp, hand
C De Simone [16]	59	Man	CML	1	N/A	N/A	Multiple	SCC	Scalp
N Aste [17]	60	Man	CML	N/A	3	10	Multiple	SCC	Face, scalp, hand
G E Pamuk [12]	73	Man	CML	1.42	3	4	Multiple	AK, SCC	Face, ear
Carla Sanchez‐Palacios [11]	69	Man	ET	N/A	7	9	Multiple	AK, SCC	Forearms, face, ear, hands
Carla Sanchez‐Palacios [11]	66	Man	CML	N/A	9	13	5 SCC, 1 BCC, multiple AK	SCC, BCC, AK	Scalp
M De Benedittis [18]	66	Man	PV	N/A	15	18	One	SCC	Tongue
R Saraceno [19]	81	Man	CML	1	0.5	5	Multiple	AK, SCC	Face, extremities
A H Kalajian [20]	82	Woman	MDS	N/A	12	15	One	SCC	Hand
Jelena Radić [21]	76	Woman	PV	N/A	N/A	14	One Multiple	BCC, SCC AK	Hand
T M Schleußinger [22]	80	Woman	ET	0.75	11	13	Multiple	Bowenoid AK, Bowen	Face
E. Antonioli [23]	65	3 Women, 4 men	5 PV, 1 ET, 1 PMF	1	N/A	N/A	Multiple 3 SCC	AK SCC	N N/A
E. Antonioli [23]	66	2 Men 1 woman	2 PV 1 ET	0.5	5	N/A	1	BCC	N N/A
Tamar Stone [24]	62	Woman	PV	1	9	10	1	SCC	heel
Neill Brett [25]	67	Man	PV	1–1.5	N/A	14	Two SCC multiple AK	SCC AK	Ear hand
T.Gambichler [26]	85	Man	PV	N/A	N/A	3	Multiple	SCC	Scalp
M.Lewandowski [27]	56	Man	PV	1.5–2 to 3	N/A	N/A	2 2	SCC AK	N/A
O.Kerdoud [28]	59	woman	ET	0.5	N/A	N/A	One	SCC	Face
Yan Xu [29]	67	woman	PMF	1	N/A	N/A	Multiple	SCC	Hands, legs
Patricia J.M.Best [3]	59	woman	ET	N/A	6.5	8	Multiple multiple one	SCC AK BCC	Face, hand, chest
	50	woman	CML	2.5	4.5	5	1 multiple	BCC SCC	Face, hand
Hilary Brown [30]	84	woman	ET	0.5	17	18	Multiple	AK SCC	Face, hand
Carmen Cantisani [10]	77	One woman 8 men	PV	N/A	N/A	N/A	Multiple	AK BCC SCC	Scalp, face, trunk
Esteve E [31]	83	Woman	PV	1	N/A	13	Multiple	SCC AK	Hand, oral
Garcia‐Martinez [32]	67	Man	PV	2	N/A	12	One	SCC	Ear
C.Vassallo [33]	70	Man	CP	0.5–2	N/A	5.75	One	SCC	Face
	55	Man	BP		N/A	6	One	SCC	Ear
	50	Man	BP		N/A	5	One	SCC	Lower lip
Viktor Simeonovski [34]	52	Man	ET	1.5	N/A	> 10	Two	BCC, AK	Brachial, face
Papi [8]	70	Man	CML	0.7–1	N/A	4	Multiple	AK, SCC, BCC	Face
Angeli [6]	67	Man	CML	1 mg/kg	2	4	Multiple	AK, SCC	Scalp
Grange [35]	60	Woman	CML	1–2	6	8	Multiple	AK	N/A
Callot‐Mellot [7]	64	Man	ET	N/A	N/A	5.5	N/A	N/A	N/A
	72	Man	PV	N/A	N/A	7	N/A	N/A	N/A
	69	Woman	PV	N/A	N/A	8.5	N/A	N/A	N/A
	76	Woman	ET	N/A	N/A	10	N/A	N/A	N/A
	76	Woman	ET	N/A	N/A	2	N/A	N/A	N/A
Salmon‐Ehr [36]	73	Man	PV	N/A	N/A	10	Multiple	AK, SCC	N/A
Wen ly [37]	55	Woman	PMF	1	8	N/A	Multiple	SCC	Hand, leg
Hoff [38]	68	Woman	PV	N/A	8	N/A	Multiple, one	AK, SCC	Leg
Elisa Zaccaria [39]	70	Man	CML	1	5	12	5	SCC	Face
Hao [40]	45	Man	CML	0.4–0.8	N/A	11	One	SCC	Hand
Wang [41]	66	Woman	CML	2–2.5	N/A	5	One	SCC	Hand
Shakoei	54	Woman	ET	1.5	12	14	Multiple	AK, Bowen, SCC	Face, hands, legs

Abbreviations: AK, actinic keratosis; BCC, basal cell carcinoma; BP, blastic phase of chronic myeloid leukemia; CML, chronic myeloid leukemia; CP, chronic phase of chronic myeloid leukemia; ET, essential thrombocytopenia; MDS, myelodysplastic syndromes; PMF, primary myelofibrosis; PV, polycythemia vera; SCC, squamous cell carcinoma.

They received the HU therapy for 9.17 ± 4.29 years (range = 2–18 years). The latency period to symptoms in patients with HU‐related skin cancer was 1–17 years (7.33 ± 4.40). The HU dose in these patients ranged from 1 to 3 g/d (1.21 ± 0.53). Among NMSCs, SCCs are more frequently associated with the HU treatment, followed by AKs, BCCs, and Bowen. Thirty‐six patients had SCC, among whom 40% had more than one lesion.

Eight patients had skin lesions clinically identified as BCC, with two cases having more than one lesion. Seven patients had Bowen, and 20 patients had AK, with lesions often located on the face and hands (*n* = 17; 28.3%). Seven patients (11.7%) had lesions on the scalp, five and six patients (8.3% and 10%) had lesions on the ear and legs, and one had a lesion on the tongue.

If NMSC or any squamous dysplasia of the skin is noticed, HU must be discontinued and replaced with an alternative medication [22]. In many cases, discontinuation of HU therapy is not feasible due to the critical role HU plays in managing myeloproliferative disorders.

Ruxolitinib is a JAK1/JAK2 inhibitor that has been proposed as an alternative treatment in HU‐intolerant or resistant PV patients with lower thromboembolism risk. This meta‐analysis significantly improved Myeloproliferative Neoplasms‐Symptom. However, patients with PV treated with ruxolitinib had higher rates of NMSCs [42]. In patients on ruxolitinib, the incidence rate of NMSC was 17.1% vs. 2.7% in the control group on the optimal medical therapy [43]. Most SCCs appeared between the first and second year after starting ruxolitinib, with a median duration of 66.5 weeks [32]. So, a multidisciplinary approach involving dermatologists and hemato‐oncologists is essential to effectively tailor treatment plans for HU therapy patients [44].

However, if HU must continue, a low dose of oral retinoid is recommended because of its chemopreventive role [45]. These patients must also have long‐term follow‐up since NMSC may recur several years after the HU withdrawal [13].

The HU withdrawal, tumor surgery, and ablation were performed. However, in some patients, including our case, lesions remained after the HU withdrawal; even new lesions developed because the cumulative effect of the HU remains for years after it is discontinued.

SCC treatment options include surgical excision, topical Imiquimod 5%, topical 5‐fluorouracil, topical diclofenac, radiotherapy, and photodynamic therapy (PDT) [30].

PDT is considered one of the several alternative therapies available for the treatment of SCC [46]. However, sometimes PDT can be combined with Imiquimod, which can increase the number of CD8 + T cells, so that it may have an enhanced effect on the treatment of SCC [47]. Other therapies like cryotherapy or chemotherapeutic (5‐fluorouracil, methotrexate, diclofenac) agents inhibit some molecules implicated in the carcinogenic process and can be combined with PDT [48, 49]. Cemiplimab is a monoclonal antibody used for SCC, blocking the PD‐1/PD‐L1 pathway. This drug is used to treat multiple SCCs in a 70‐year‐old female patient suffering from ET. After six cycles, erythema and desquamation decreased [50]. Sometimes, Cemiplimab (an anti‐PD‐1 antagonist) is co‐administered with interferon alfa‐2b (peginterferon) to control both SCC and PV in a patient with SCC aggravation following ruxolitinib exposure [51].

UV protection, regular dermatological evaluations, and biopsy of any suspicious lesions are necessary for this type of patient [44].

## 4 Conclusion

Increasing awareness about the side effects of HU therapy is necessary, and patients treated with long‐term HU must undergo regular dermatological assessments. Regular skin examinations and patient education regarding the signs of skin changes are essential components of care for individuals on long‐term HU therapy. Moreover, developing guidelines for monitoring and managing skin health in this population is warranted to mitigate the risk of NMSCs.

## Author Contributions

**Safoura Shakoei:** study concept, design, and technical supervision, acquisition of data and drafting of the manuscript, critical revision of the manuscript. **Zahra Nilfroushan:** acquisition of data and drafting of the manuscript and writing – original draft preparation.

## Disclosure

The authors have nothing to report.

## Consent

After a complete explanation of the report's purpose, written informed consent was obtained from the patient.

## Conflicts of Interest

The authors declare no conflicts of interest.

## Data Availability

The data that support the findings of this study are available from the corresponding author upon reasonable request.
